# Genotypic Differences in Networks Supporting Regional Predictors of Speech Rate in Spinocerebellar Ataxia: Preliminary Observations

**DOI:** 10.1089/brain.2020.0972

**Published:** 2021-08-10

**Authors:** John J. Sidtis, Christopher M. Gomez

**Affiliations:** ^1^ Brain and Behavior Laboratory, Geriatrics Division, The Nathan Kline Institute for Psychiatric Research, Orangeburg, New York, USA.; ^2^ Department of Psychiatry, New York University Langone Medical Center, New York, New York, USA.; ^3^ Department of Neurology, The University of Chicago, Chicago, Illinois, USA.

**Keywords:** ataxia, cerebellum, dysarthria, functional imaging, speech, spinocerebellar ataxia

## Abstract

***Background:*** Disordered speech production, dysarthria, is a common characteristic of the spinocerebellar ataxias (SCAs). Although dysarthric features differ across SCAs, a previous analysis revealed that a combination of regional cerebral blood flow (rCBF) in the left inferior frontal region and the right caudate predicted syllable rate, a pattern reported in normal speakers. This study examined the relationships between primary predictor brain regions and other areas of the brain in three SCA groups. The regions associated with the primary predictors are considered as elements of secondary networks since they are associated with regional speech predictors rather than directly with speech performance.

***Methods:*** Speech and rCBF data from 9 SCA1, 8 SCA5, and 5 SCA6 individuals were analyzed. Partial correlations were used to identify brain regions associated with the primary predictors.

***Results:*** Secondary networks differed across SCA genotypes. SCA1 and SCA6 demonstrated both positive and negative associations between primary and secondary areas, whereas the associations in the SCA5 genotype were only positive. The SCA5 associations were also largely bilaterally symmetrical. Both SCA1 and SCA5 demonstrated secondary associations with the right caudate, whereas the SCA6 group had no such associations.

***Conclusions:*** These results demonstrate that although primary aspects of a brain network may remain functional, pathophysiological processes associated with different SCA genotypes may express themselves in alterations of broader, secondary brain networks. These secondary networks may reflect generic functional associations with the primary predictor regions, compensatory activity in the presence of an SCA, SCA pathology, or some combination of these factors.

**Impact statement:**

This study demonstrates that although the primary predictors of speech rate in the brain are shared in normal speakers and three genotypes of ataxia, the genotypes differ from each other in broader activity patterns associated with the primary predictors. One implication is that although basic neural circuitry may remain functional for some period of time in progressive neurological disorders, abnormal relationships may exist in the broader neurological context in which they operate. These results also serve as a reminder that patterns of brain activity reflect involvement in the external world as well as the brain's response to itself.

## Introduction

The spinocerebellar ataxias (SCAs) have common features, including impaired gait and limb coordination and disorders of speech production, ataxic dysarthria (Schalling and Hartelius, 2013). With the identification of specific genotypes associated with specific hereditary ataxias, a broader range of signs and symptoms have been recognized with different SCAs. More than 40 genotypes have currently been identified (Coarelli et al., 2018; Sun et al., 2016). This study focuses on three of these (SCA1, SCA5, and SCA6), whose members were studied with speech examinations and Positron Emission Tomography (PET) scans of regional cerebral blood flow (rCBF) while speaking (Sidtis et al., 2006, 2010).

Speech characteristics differ among these SCAs (Sidtis et al., 2011) but using a performance-based analysis (PBA) of rCBF response connectivity obtained with PET (Sidtis 2012a; Sidtis et al., 2003, 2007), a simple, reproducible, clinically relevant network associated with speech rate has been established. SCA1, SCA3, and SCA5 share the feature of having their speech rates predicted by an rCBF pattern that consists of an increase in the left inferior frontal region coupled with a decrease in the head of the right caudate nucleus as speech syllable repetition rates increase. This pattern of primary predictive regions was originally observed and subsequently replicated in normal speakers (Sidtis et al., 2003, 2018b). Relevant white matter connections between these functionally associated brain regions also appear to be sensitive to some of the physical characteristics of the acoustic speech signal (Sidtis et al., 2018a) supporting these relationships.

The cortical–subcortical primary predictors of syllable rate in the SCA subjects included two additional brain regions not identified in the normal pattern: an increase in right cerebellar rCBF and a decrease in the left transverse gyrus rCBF (Sidtis et al., 2006). These rCBF associations with speech production are consistent with lesion studies, which have associated the right cerebellum with speech control in right-handed subjects (Ackermann et al., 1992; Amarenco et al., 1993; Urban et al., 2001, 2003), a relationship consistent with crossed cortical–cerebellar diaschisis (Carrera and Tononi, 2014; Pantano et al., 1986). With respect to the left temporal region rCBF association, it was suggested that changes in the left temporal region may indicate an altered role for auditory feedback during ataxic speech (Parrell et al., 2017; Sidtis et al., 2006).

In the SCAs studied with PET, the primary predictive cortical–subcortical pattern persisted over the course of 2 years of disease progression (Sidtis et al., 2010). Overall, rCBF was reduced at the second evaluation, with greater reductions in right-sided compared with left-sided regions. With respect to the longitudinal changes in the primary predictor regions, left inferior frontal rCBF increased significantly whereas right inferior frontal rCBF decreased between the first and second evaluations. This observation is inconsistent with the suggestion that homotopic compensation plays a role in language recovery after damage to a traditional language area. The caudate nucleus did not reflect significant lateralized rCBF changes over time. Cerebellar rCBF declined bilaterally at the second evaluation.

In normal speakers, we have extended the performance-based analysis (PBA) by examining the relationships among the primary speech predictor cortical region (left inferior frontal) and basal ganglia regions (head of the right caudate nucleus) with rCBF in other brain areas (Sidtis, 2012a). This extension of the PBA acknowledges the likelihood that even a reliable predictive network of a small number of brain regions represents the “tip of the iceberg” of a larger neural system responsible for the complex behaviors of speech and language. The present study focuses on this second stage of PBA to examine the brain regions associated with the primary predictors in SCA subjects. The existence and identities of additional brain regions associated with the primary predictors would represent a neurophysiological context in which the primary predictors function. As such, although the three SCA genotypes studied shared the same primary predictors as normal speakers, differences in neurophysiological contexts in which they function may reflect differences in the effects of each SCA pathophysiology.

## Methods

### Participants

This study was approved by the institutional review boards for studies involving human subjects at the University of Minnesota and the Minneapolis Veterans Affairs Medical Center. After study procedures and their possible consequences were explained, all subjects provided informed consent to the protocol according to standards established by the Declaration of Helsinki and approved by the Institutional Review Boards of the University of Minnesota Medical School and the Minneapolis Veterans Affairs Medical Center.

The present study is a retrospective analysis of PET data originally collected as part of an NIH funded program to study familial SCA. Family members from clinically identified genotypes were brought to the University of Minnesota for several days of clinical, laboratory, and imaging studies. The age range of participants was 18 to 70 years. The ataxia participants have been previously described (Sidtis et al., 2006). A group of 22 volunteers with hereditary ataxia participated in this study. Seven of these subjects were studied twice over an average period of 1.9 years. There were six male and three female SCA1 subjects (Orr et al., 1993; average trinucleotide repeat size 50.9; range 45–59), two male and six female SCA5 subjects (Ranum et al., 1994; non-nucleotide repeat disease), and five male SCA6 subjects (Du et al., 2013; Gomez et al., 1997; Zhuchenko et al., 1997; average trinucleotide repeat size 22.4; range 22–23). The mean ages (±standard deviation) for each group were: SCA1 34.8 ± 16.1 years; SCA5 45.9 ± 16.3 years; and SCA6 51.2 ± 15.8 years.

All participants were right-handed, native speakers of English. Subjects were screened to exclude confounding neurologic, psychiatric, and medical disorders, and to exclude current psychotropic medication or recreational drug use. None were treated pharmacologically for ataxia. All SCA participants were found to have some degree of cerebellar atrophy on clinical magnetic resonance imaging (MRI) studies, but quantitative morphological data were not available (Sidtis et al., 2006).

The means and ranges of ratings from each SCA group on a neurological examination standardized for the program project are presented in Table 1. The evaluation of participants in the project preceded the development of standardized examinations such as the Scale for the assessment and rating of ataxia (SARA; Schmitz-Hübsch et al., 2006) but each participant was evaluated by a neurologist (C.M.G.) highly experienced in examining ataxic patients. Previously published data from normal subjects (eight female, five male right-handed native English speakers, mean age 43 ± 11 years) are included for reference (Sidtis et al., 2003; Sidtis, 2012a).

**Table 1.** Summaries of Features from the Standard Neurological Examinations for the Spinocerebellar Ataxia Groups in this Study

	Upper limb coordination		Lower limb coordination	Gait and station	
Group	F-to-N	R-A-M	Heel-Shin	Truncal stability	Gait base
SCA1 severity rating	1.1	1.3	0.6	0.7	1.2
SCA1 severity range	0–3	0–3	0–2	0–2	0–4
SCA5 severity rating	0.5	1.3	1.2	0.8	0.8
SCA5 severity range	0–2	0–3	0–3	0–2	0–3
SCA6 severity rating	0.8	1.6	1.2	0	0.8
SCA1 severity range	0–2	0–3	0–2	0	0–3

Mean scores and ranges for each group are provided. For the finger-to-nose (F-to-N) evaluation: 0 = normal, 1 = mildly inaccurate, 2 = mild tremor at endpoint, 3 = consistent tremor throughout movement. For the rapid alternating movement (R-A-M) of the hand: 0 = normal, 1 = slow, 2 = irregular, 3 = slow and irregular. For the movement of the heel of the foot down the shin (Heel-Shin): 0 = normal, 1 = mild dysmetria with or without end tremor, 2 = moderate dysmetria (severe tremors before reaching the ankle), 3 = severe dysmetria (cannot place the ankle on the knee). Most abnormalities were bilateral, but the more severely affected side provided the rating. Truncal stability was scored as follows: 0 = normal, 1 = mild to moderate oscillations of head/trunk, 2 = severe oscillation, 3 = unable to sit without support. Gait base was scored as follows: 0 = normal, 1 = mild widening to about 4–10 cm apart, 2 = moderate widening to about 10–30 cm apart, 3 = severe widening to >30 cm apart. These individuals were evaluated prior to the introduction of the SARA scale for the assessment and rating of ataxia (Schmitz-Hübsch et al., 2006).

SARA, scale for the assessment and rating of ataxia; SCA, spinocerebellar ataxia.

### Scanning conditions

Subjects were studied with eyes covered, room lights dimmed, and earphones placed in foam headrests custom-made for each subject. Subjects were required to remain awake, quiet, and still during each scan. The speech task consisted of the repetition of the syllable sequence/*pa-ta-ka/*, produced as quickly as possible as is performed in standard diadochokinesis motor speech examinations (Kent et al., 1987). Subjects were instructed to take a deep breath, and then to produce as many syllables as possible during expiration. Subjects repeated this process for 60 sec during the scanning period. Syllable repetitions were audio-recorded during the scans for subsequent analyses.

### Behavioral measures

Speech recorded during each scan was used to determine syllable repetition rates. The number of syllables produced during the initial 60 sec of each PET scan acquisition were counted from the recorded utterance and divided by the production time. Pauses were not subtracted for this calculation. Dysarthria ratings were assigned by two Master's Degree level speech pathology interns based on speech samples recorded during a speech examination performed before each scanning session. There was a high degree of agreement between the two raters. When differences occurred, they were resolved by a consensus meeting with a senior speech pathologist. The dysarthria scores had a possible range of 1 (mildest) to 9 (most severe). Table 2 presents the mean syllable rates (syllables per second) produced during PET scanning, and clinical dysarthria severity ratings for each SCA subject determined from the speech examination performed before scanning. The dysarthria rating scores represented perceptual judgments by speech pathologists, with a possible range of 1 (mildest) to 9 (most severe; Sidtis et al., 2006).

**Table 2.** Group Mean Values (Standard Deviations) for Syllable Repetition Rates During Scanning and Spinocerebellar Ataxia Clinical Dysarthria Ratings Based on Clinical Examinations

Measure	Normals	SCA1	SCA5	SCA6
Syllables per second	4.0 (0.6)	3.6 (1.1)	3.6 (1.0)	3.4 (1.0)
Dysarthria rating	—	2.3 (1.8)	2.1 (1.1)	3.0 (1.9)

### PET scanning

Bolus injection of [^15^O] water was used as a marker of rCBF (Silbersweig et al., 1993). Each study consisted of eight 90-sec scans (four rest scans alternating with four speech scans), separated by an inter-scan interval of ∼9 min, acquired by using a Siemens-ECAT 953B tomograph in 3D mode (Sidtis et al., 1999).

The subjects were told to start 10 to 15 sec before estimated isotope detection by the scan and asked to stop after 60 sec. The time delay was measured during an initial O15 injection before the initiation of the speech protocol. This was used for estimating delay times and the speech starting times for the remainder of the scans. The typical delay times were between 40 and 50 sec.

### Functional imaging measures

A set of 22 standard regions-of-interest (ROIs) was used for this protocol (Sidtis et al., 1999, 2003, 2006). The original region templates were drawn on composite images that consisted of multiple scans to improve resolution. Co-registered MRI was not used in this process. For each subject, the templates were first applied to a composite image of all the speech images obtained for that subject. Minor adjustments for individual anatomical differences were made for each subject's template when necessary. An individual's templates were then applied to each of the four speech scans. As the PET images were not registered to a standard atlas, small adjustments were made for differences in brain volume and head tilt. Since the data for each ROI were subject to thresholding, small adjustments in the size and position of each ROI were not critical as the data were sampled at an upper 25% threshold rather than representing the entire area of the ROI. This minimizes boundary errors.

The ROI approach is fundamentally different from a voxel-based approach. Each ROI captured an anatomical region (e.g., inferior frontal region) without attempting to draw specific anatomical boundaries. Within each ROI, a threshold was applied such that ROI values represented the mean of the upper 25% of voxel values (Rottenberg et al., 1991; Sidtis et al., 2003). This approach has two benefits. Thresholding reduces the partial volume error in measuring blood flow in the mixtures of gray and white matter that usually occurs within the resolution of functional imaging. More importantly, the ROI approach tolerates normal intra-subject variation in the locus of the thresholded voxels within an anatomical region.

The voxel-based requirement for considering common voxels across subjects can be avoided with the ROI approach (Sidtis 2012b). This is important because although many warping algorithms spatially normalize individual images to yield acceptable approximations to a standard template brain, there are no distortion free methods that accommodate the individual differences in anatomy (Toga and Thompson, 2002). Resting-state scans were not used in this analysis, as it has been shown that rCBF in this condition is influenced by task conditions in scans with which they are paired (Sidtis et al., 2004).

The following regions (left and right) were examined: inferior, mid (including the dentate nucleus), and superior portions of cerebellum in horizontal planes, superior temporal gyrus (Brodmann's area 22), transverse temporal gyrus (Brodmann's area 41, Heschl's gyrus), putamen, caudate nucleus (head), thalamus, inferior frontal lobe (Brodmann's areas 44 and 45, including Broca's area), sensorimotor cortex (Brodmann's areas 3 and 4), and supplementary motor area (Brodmann's area 6). The set of ROIs was applied interactively to the images for each subject, allowing the ROIs to be slightly modified to conform to individual differences.

The mean global activity (all voxels) from each whole-brain scan was used to create a normalized measure of rCBF. The normalization factor for each subject was the ratio of the highest volume mean in the dataset meeting the upper 25% threshold and the individual subject's volume mean. Each subject's ROI values were then multiplied by his or her normalization factor. The normalized rCBF reduces irrelevant inter-subject variability due to global differences (Arnt et al., 1996; Sidtis et al., 2003). The ROIs were not subdivided into smaller sub-units (e.g., thalamic divisions, sensory vs. motor strips), as examinations of rCBF volumes before extracting ROIs did not provide any obvious separations supporting finer classifications.

### Statistical analyses

**FIG. 1.**
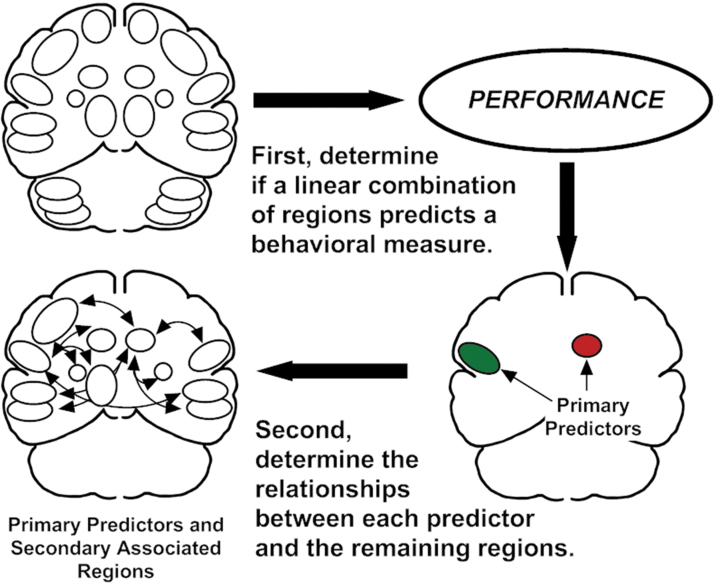
A schematic description of PBA. Rather than employing subtraction or other form contrast between scanning conditions, a multiple linear regression with a stepwise selection process is used to determine a relationship between rCBF and specific behaviors measured during scanning. The first stage of this process has identified two primary regions that interact to predict speech syllable repetition rates in normal (Sidtis 2012a; Sidtis et al., 2003, 2018b) and SCA speakers (Sidtis et al., 2006; 2010). In the second stage of PBA, the relationships between the primary predictor regions and each of the other regions in the data set are determined by using partial correlations, controlling for the effects of the predictor region's homologous area in the opposite hemisphere. PBA, performance based analysis; rCBF, regional cerebral blood flow; SCA, spinocerebellar ataxia.

Multiple linear regression analysis (SPSS, 1988) was originally used to determine the primary rCBF regions that predicted syllable production rates (Sidtis et al., 2003, 2006). This is the first stage of the Performance-Based Analysis (PBA) depicted in Figure 1. In addition to speech rate, this approach has been used to identify rCBF patterns associated with vowel stability (Sidtis, 2015) and propositional and formulaic language modes (Sidtis et al., 2018b; Van Lancker Sidtis and Sidtis, 2018a, 2018b) in spoken utterances. It has also revealed an abnormal rCBF pattern during Parkinsonian speech and the partial normalization of this pattern during speech after stimulation of the subthalamic nucleus (Sidtis et al., 2021).

The second phase of this analysis added a new element of this approach. Partial correlation procedures were used to investigate possible relationships between the two primary predictor regions and the remaining regions in the data set (Sidtis, 2012a). The partial correlation technique was used to control for the influence of the contralateral region homologous to a primary predictor, as left/right homologous regions tend to be positively correlated. The partial correlation technique employs a Pearson correlation procedure after accounting for the effects of the homologous ROI. The approach to this analysis was based on establishing simple relationships, as it was believed that the influences on blood flow changes were multifactorial. The 90 sec acquisition periods for the PET data would not be useful in establishing a temporal directionality of these relationships.

This is a descriptive study that explores the potential value of using a new technique to examine the functional context in which specific brain regions are associated with specific behaviors. As partial correlations between each of the two primary predictor regions for speech rate were examined for the remaining 20 other regions for each genotype, the issue of multiple comparisons was addressed. To address this issue, the conservative Bonferroni correction was used to provide criteria for significance. A probability value had to be less than 0.0025 to be reported in the partial correlation results. Independent-group *t*-tests were used to compare some group differences and Fisher's exact tests were used to evaluate the relative proportions of secondary association regions with each of the primary predictor regions, as well as the relative proportions of positive and negative secondary associations with each of the primary predictor regions.

## Results

Although the three SCA genotypes had some differences in neurological motor signs (Table 1), the rated abnormalities varied within the mild range in each genotype. Their levels of abnormal performance on the speech task were comparable. Each of the SCA groups produced significantly fewer syllables per second than the normal reference subjects: [SCA1: *t*(97) = 2.52; *p* = 0.01; SCA5: *t*(105) = 2.58; *p* = 0.01; SCA6: *t*(68) = 3.11; *p* = 0.003]. This was consistent with the previously reported clinical ratings for diadochokinesis in SCA (Ackermann et al., 1992; Schalling and Hartelius, 2004). The SCA groups did not significantly differ from each other in either their rates of syllable productions during scanning or their clinical dysarthria ratings (Table 2). In the formal speech examination as well as during the interactions over the week-long study period, clinical judgments of strained-strangled voice (a possible marker of spasticity) was not uniformly noted across different speaking contexts and was rarely rated as clinically significant (Sidtis et al., 2011). None of the subjects were treated for ataxic signs or symptoms, including speech spasticity.

The partial correlation analysis was used to identify brain regions associated with the two primary predictors. For the left inferior frontal region, where activity increased with increased speech rate, a positive relationship with another brain region indicated that the other region increased activity with the inferior frontal region. A negative relationship with another brain region indicated that the other region decreased activity as the inferior frontal region increased.

For the right caudate, the pattern was different. Because the right caudate activity decreased with increased speech rate, a positive relationship with another brain region indicated that the other region decreased activity as the right caudate decreased. A negative relationship between the right caudate and another brain region indicated that the other region increased activity as the right caudate decreased.

Table 3 presents the secondary relationships between the left inferior frontal region and the remaining brain regions during syllable repetitions. The number of partial correlations differed across groups. For the normal reference group, there were three relationships: two positive, one negative. For the SCA1 group, there were three relationships: two positive, one negative. In the SCA6 group, there were six relationships: Four were positive, and two were negative. However, the SCA5 group had 16 relationships: all positive.

**Table 3.** Partial Correlation Values (and Significance Levels) Between the Left Inferior Frontal Gyrus Region (Controlling for the Influence of the Right Inferior Frontal Gyrus Region) and the Other Regions in the Data Set During Speech Syllable Repetition

Region	Normals	SCA1	SCA5	SCA6
L INF CBL				
R INF CBL			+0.411 (*p* = 0.002)	
L MED CBL			+0.568 (*p* < 0.001)	
R MED CBL			+0.506 (*p* < 0.001)	+0.829 (*p* < 0.001)
L SUP CBL				+0.667 (*p* = 0.002)
R SUP CBL				+0.785 (*p* < 0.001)
L STG		+0.456 (*p* = 0.002)	+0.868 (*p* < 0.000)	
R STG		+0.633 (*p* < 0.001)	+0.7 (*p* < 0.001)	
L TTG		−0.47 (*p* = 0.001)	+0.703 (*p* < 0.001)	
R TTG			+0.776 (*p* < 0.001)	
L PUT	+0.5 (*p* < 0.001)		+0.87 (*p* < 0.001)	
R PUT			+0.865 (*p* < 0.001)	
L CAU	+0.482 (*p* < 0.001)		+0.808 (*p* < 0.001)	+0.819 (*p* < 0.001)
R CAU			+0.764 (*p* < 0.001)	
L THA	−0.49 (*p* < 0.001)		+0.702 (*p* < 0.001)	
R THA			+0.676 (*p* < 0.001)	−0.687 (*p* = 0.001)
L IFG				
R IFG				
L SMS			+0.608 (*p* < 0.001)	
R SMS			+0.526 (*p* < 0.001)	−0.644 (*p* = 0.003)
L SMA				
R SMA			+0.55 (*p* < 0.001)	

Only correlations for which *p* < 0.0025 are reported (Bonferroni correction).

IFG, inferior frontal gyrus; SMA, supplementary motor area; SMS, sensory-motor strip; STG, superior temporal gyrus.

The secondary relationships between the right caudate and the other regions are presented in Table 4. The normal reference group had five relationships: four positive, one negative. For the SCA1 group, there were seven relationships: three positive, four negative. The SCA6 group had no secondary relationships involving the right caudate. The SCA5 group had six relationships. As was the case with the inferior frontal region, all SCA5 secondary relationships were positive.

**Table 4.** Partial Correlation Values (and Significance Levels) Between the Right Caudate Region (Controlling for the Influence of the Left Caudate Region) and the Other Regions in the Data Set During Speech Syllable Repetition

Region	Normals	SCA1	SCA5	SCA6
L INF CBL				
R INF CBL				
L MED CBL				
R MED CBL				
L SUP CBL				
R SUP CBL				
L STG	+0.514 (*p* < 0.001)	+0.584 (*p* < 0.001)	+0.409 (*p* = 0.002)	
R STG	+0.598 (*p* < 0.001)		+0.537 (*p* < 0.001)	
L TTG		−0.47 (*p* = 0.001)		
R TTG				
L PUT		+0.531 (*p* < 0.001)	+0.452 (*p* = 0.001)	
R PUT	+0.661 (*p* < 0.001)	+0.582 (*p* < 0.001)	+0.668 *(p* < 0.001)	
L CAU				
R CAU				
L THA				
R THA				
L IFG			+0.418 (*p* = 0.002)	
R IFG	+0.531 (*p* < 0.001)		+0.437 (*p* = 0.001)	
L SMS	−0.524 (*p* < 0.001)	−0.452 (*p* = 0.002)		
R SMS				
L SMA		−0.541 (*p* < 0.001)		
R SMA		−0.606 (*p* < 0.001)		

Another difference across SCAs was the bilaterality of the secondary relationships. In the normal group, there were a total of eight secondary relationships with only one region involved in a bilateral relationship (a positive association). For the SCA1 group, there were a total of 10 secondary relationships, 2 of which were bilateral (1 positive, 1 negative). In the SCA6 group, there were a total of six relationships with one being bilateral (a positive association). There were 22 secondary relationships in the SCA5 group, with 10 regions being bilaterally involved (positive relationships).

## Discussion

The results of this study are based on patterns of correlations between rCBF in each of the two brain regions that reliably predict speech rate and other areas of the brain. The goal of this study was to determine whether the predictors of speech rate functioned in different neurophysiological contexts (secondary relationships) in the three SCAs studied. The rCBF data for each genotype were analyzed in the same way by using the same criteria. Nevertheless, the patterns of secondary relationships differed across the three SCAs. The SCA1 groups had 10 secondary relationships. The SCA6 group had six secondary relationships, but none involved the right caudate. The SCA5 group had 22 secondary relationships, with 10 of these relationships demonstrating bilaterality. In contrast, bilaterality was present in only one region in the normal group, and in two regions in the SCA1 and SCA6 groups.

Unlike the normal, SCA1, and SCA6 groups, all secondary relationships were positive in SCA5. Both SCA1 and SCA6 are trinucleotide repeat disorders, whereas SCA5 is not. The results of this study are necessarily descriptive, but the patterns of secondary relationships appear to be qualitatively different across the SCAs. These differences raise fundamental issues about the nature of patterns of functional brain activity and the levels at which such activity is affected by genotype in SCA.

The different SCAs have been characterized with respect to the relative saliences of pyramidal and extrapyramidal signs (Sun et al., 2016) as well as dysarthria. A multiple linear regression analysis previously identified the ratings of the speech characteristics of individuals in these three genotypes (Sidtis et al., 2011). Speakers in each genotype showed slowing of rapid alternating movements (fewer syllables/sec) and imprecise consonants. Excess and equal stress in speech and irregular articulation were more important in identifying individuals with SCA1, but prolonged phonemes were not a factor that distinguished SCA1. Strained-strangled voice, which reflects vocal spasms but has been characterized as the result of laryngeal dystonia, was not identified as an identifying factor in SCA6. Harsh voice was not identified as a predictive characteristic of these SCA5 individuals who had no extrapyramidal signs.

Qualitatively, SCA1 had the more relatively normal pattern of positive and negative secondary relationships with the left inferior frontal gyrus and the right CAU and had predictive subjective ratings in each speech characteristic examined, except prolonged phonemes. For SCA5, which had atypical, uniformly positive secondary relationships, neither harsh voice nor irregular articulation was an identifying characteristic. SCS6 was also atypical, with no secondary relationships with the right CAU. Neither strained-strangled nor irregular articulation voice ratings were predictive of identification with this SCA. With the respective complexities of speech motor control and neurological networks, the present work clearly represents a preliminary effort. However, it does suggest that identifying relevant brain–behavior relationships should take into account behavior, primary brain network responses, and the neurological environment in which such responses are occurring.

These results do not address specific roles for the secondary regions identified in this study, but each region has been implicated in some aspect of vocalization, speech, or language. This brief review will focus on data from lesion or neurosurgical brain stimulation studies rather than other functional imaging studies. Functional imaging has increasingly gravitated toward complicated signal processing of large data sets, yielding results that frequently have no clear relationship to well-established clinical data. Modern imaging techniques are sensitive at capturing signals in the brain, but the interpretive difficulty derives from several factors. Cerebral blood flow measurements at any anatomical point represent the activity of a system referred to as a neurovascular unit, which represents the actions of different types of blood vessels, vasoactive agents, and neural cell types (Drake and Iadecola, 2007). On a functional level, all signals do not represent the same computational or executive functions in complex neurological systems (Sidtis et al., 2003).

Given the inherent complexity of the basis of functional imaging signals, we have suggested that conclusions about regional brain activity should rely on more than one source of information to maintain a clinical perspective on functional imaging results (Sidtis et al., 1996).

Penfield (1938) reported the results of electrical stimulation of the exposed brains of six patients. At various places along the sensorimotor strip, bilaterally, especially in premotor areas, stimulation produced vocalizations of vowel sounds. Varying the stimulation parameters and locations produced changes in the amplitudes, pitches, and vowel articulation of the involuntary vocalizations. Words were never elicited. Penfield and Rasmussen (1949) reported similar results for an additional 29 cases and speech arrest followed a similar anatomical mapping, with the addition of stimulation of the dominant inferior frontal region also producing speech arrest. Penfield and Welch (1951) stimulated the supplementary motor in seven patients, producing rhythmic or intermittent vowel sounds and repetitions of a vowel produced when the stimulation was introduced during speaking. Repetitive vocalizations were also reported when supplementary motor stimulation was introduced while the patient was speaking by Brickner (1940). Jonas (1981) presented 4 cases and reviewed the literature from an additional 53 published cases, with similar results. Hertrich et al. (2016) reviewed additional lesion studies identifying the supplementary motor area's involvement in speech and further suggested that speech changes in subcortical neurodegenerative diseases such as Huntington's and Parkinson's may reflect abnormal interactions between the supplementary motor area and the basal ganglia.

Ojemann et al. (1968) studied 28 patients undergoing thalamotomy. Stimulation produced disturbances of speech that primarily consisted of anomia and sensory changes, and in one instance, an inability to speak. Ojemann and Ward (1971) reviewed the case literature on the effects of thalamic lesions on speech and reported similar results in 25 additional patients. Schaltenbrand (1965) reported that thalamic stimulation yielded “compulsory speech,” including monosyllabic yells, exclamations, and even full utterances.

Although the temporal lobes are typically associated with speech perception, damage in this region is also associated with deficits in productive speech and language. Borovsky et al. (2007) used voxel-based-lesion-symptom mapping, identifying various regions of the temporal lobe associated with expressive and receptive language disorders and anomia. Temporal lobe regions are connected with anterior language areas in the inferior frontal lobe via the arcuate fasciculus. After stroke, the extent of damage to the arcuate fasciculus predicted the rate, informativeness, and efficiency of speech (Marchina et al., 2011). During neurosurgical procedures to resect tumors, electrical stimulation of the arcuate fasciculus produced speech arrest, inability to speak, and phonemic and semantic paraphasias (Leclercq et al., 2010).

The involvement of the basal ganglia (Caplan et al., 1990) and the cerebellum (Ackermann et al., 1992; Amerenco et al., 1993; Urban et al., 2001, 2003) in speech has been well documented in cases of neurological damage.

**FIG. 2.**
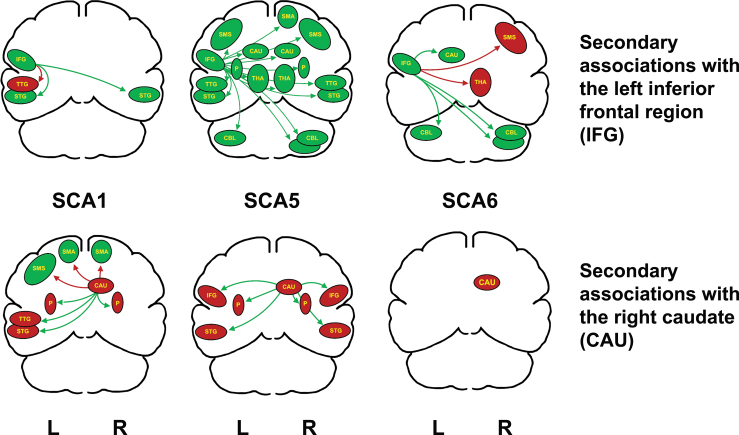
The secondary associations and the directions (positive or negative) of their correlations with the left inferior frontal region (IFG; top row) and the right head of the caudate (CAU; bottom row) for each genotype. The green arrows represent positive correlations, and the red arrows represent negative correlations. The color of each region (green representing increasing, red indicating decreasing) represents the region's relationship to the individual predictor region. The left IFG is green to indicate that rCBF increases with speech rate. Green-colored secondary regions increase with increases in IFG activity and speech repetition rates. Red-colored secondary regions decrease with increases in IFG activity and speech rate. The regions significantly correlated with the IFG activity (top row) are represented (CBL = cerebellum, three levels; STG = superior temporal gyrus; TTG = transverse temporal gyrus; THA = thalamus; P = putamen; CAU = head of the caudate; SMS = sensory-motor strip; SMA = supplementary motor area). The right caudate nucleus (CAU) is the other primary predictor of speech rate (Bottom row). Red indicates that decreased right CAU rCBF is associated with increased speech rate. As with the IFG, the green arrows indicate positive correlations with the CAU, and the red arrows indicate negative correlations. Consequently, green-colored regions (with red arrows) increase rCBF with decreased CAU activity and increased speech rate. Red-colored regions (with green arrows) decrease rCBF with right CAU decreases and increased speech rates. IFG, inferior frontal gyrus.

In a study of the speech characteristics of individuals from the three genotypes in this study (Sidtis et al., 2011), the SCA6 group was the most impaired genotype across the range of clinical dimensions studied. In Figure 2, it is the only group that did not demonstrate secondary associations with the right caudate. This suggests a weaker secondary network supporting the primary subcortical predictor in SCA 6. Feedforward and feedback mechanisms involving the temporal lobes have also been implicated in neural systems for speech (Guenther et al., 2006; Parrell et al., 2017; Spencer and Slocomb, 2007). Again, SCA6 exhibited no secondary involvement of temporal lobe regions whereas SCA1 and SCA5 demonstrated bilateral temporal lobe involvement in secondary networks with the left inferior frontal region.

The paucity of secondary associations with the left inferior frontal region in SCA1 may play a role in the abnormal voice ratings in this group (Sidtis et al., 2011). The heterogeneity of secondary associations with primary predictors across genotypes may contribute to the debate over whether the characteristics of ataxic speech reflect instability and inflexibility in production or the characteristics of failures in specific subsystems (Spencer and Dawson 2019). The ataxic brain may inherently produce speech instability and inflexibility, but differences in secondary support networks may favor some speech subsystems over others.

At least some of the secondary regions identified in this study are likely involved in more complex networks that are responsible for the variety of behaviors that fall into the category of speech. However, secondary brain regions are also likely to have a different and varied status when compared with the brain regions directly associated with speaking. In his Harvey Lecture, Penfield (1938) discussed functions of the cortex and reflected on Pavlov's view that “the entire cortex probably represents a complex system of analyzers of the internal, as well as the external, environment.”^(p420)^ Specifically, Pavlov (1927) commented on the sensitivity of cortical activity to internal as well as external events.^(p379)^ The important point is that specific behavior and its associated brain activity are not simply linked in a complex series of reflex arcs. The brain is not only responding to external stimuli and initiating behavior, but it is also responding to itself. This concept is beginning to be recognized in contemporary functional imaging (Hutchison et al., 2013).

In the framework laid out by Pavlov and Penfield, the speaking task could be considered an external demand, which was adequately met in part by the inverse relationship between the left inferior frontal region and the right caudate nucleus (the primary predictors). The regions associated with the primary predictors could be characterized as the brain's responses in support of the primary predictors (internal demand). This characterization is a reminder that any functional map of brain activity includes the brain responding to itself. Compared with the relatively normal pattern of primary region activity, the differences in the SCA patterns may represent compensation, an expression of genotypic specific dysfunction, or some combination of both. SCA6, the most speech impaired group, appears to have a more impoverished secondary network compared with that observed for the significantly less speech impaired SCA5 group.

Also relevant to the present results, Pavlov (1927) also argued that the processes of inhibition and excitation were sufficient to account for the behavioral expression of neurological function.^(p377)^ In the present study, the concepts of excitation and inhibition in secondary associations are complex, dependent on the responses of the primary predictors. In the normal, SCA1, and SCA6 groups, increases in the primary regions could be associated with either increases in secondary regions (positive relationships) or decreases in secondary regions (negative relationships). Conversely, decreases in the primary regions could be associated with either decreases in secondary regions (positive relationships) or increases in secondary regions (negative relationships).

There was no single pattern that could be characterized as excitation or inhibition for either the inferior frontal or caudate regions for these groups. The SCA5 group did not express this flexibility; all associations were positive. Each of the many secondary regions associated with the inferior frontal region increased their activity with the inferior frontal region, an effect that appears similar to excitation. Each of the secondary regions associated with the caudate region decreased their activity with the caudate region, an effect that appears to be similar to inhibition. As this is a descriptive study, these results should stimulate a re-consideration of models of brain function based solely on the notion of activation.

This study's major limitation is that it is exploratory in nature. The positive side of this is that it raises issues about primary and secondary abnormalities that accompany the hereditary ataxias that can be formalized and explicitly tested. A second limitation is the result of the modest sizes of the groups. For example, it would be interesting to determine whether changes in secondary functional brain networks are associated with trinucleotide repeat size. At the time the original project was conducted, the inclusion of several SCAs rather than a focus on a single SCA was seen as more valuable, given the resources available and the limited subject pool. It is quite feasible that future work can address these limitations.

## Conclusions

Although the current sensitivity of functional imaging technology can only identify the “tip of the iceberg” of undoubtedly more complicated neurological systems (Sidtis, 2012b), these results demonstrate the possibility that a relevant behavior (i.e., speech) in SCA may be maintained to some extent as long as the normal primary aspects of a brain network remain functional. Genotypic differences in secondary networks may be reflected in phenotypic differences. The neuropathology of the SCAs may be better reflected in alterations of broader, secondary, supportive, or reactive networks than in primary networks that can maintain a degree of normal behavior. Some of the secondary functional connections may reflect compensatory networks for controlling speech production in the context of each SCA genotype whereas some may reflect network alternations that are more reflective of each SCA pathophysiology, independent of speech.

Identifying the primary and secondary functional networks involved in symptomatic behaviors in neurogenetic progressive disorders may provide a basis for identifying general principles of functional pathologies characteristic of specific genotypes, especially when they represent pathophysiological processes (e.g., driven by trinucleotide repeats compared with other genetic abnormalities). Convergence of the patterns of secondary networks for different behaviors within a genotype may also contribute to better understanding phenotypic differences across and within genotypes (Maschke et al., 2005).
